# The Effects of an Online Patient Portal on Nurses’ and the Health Care Team's Work in an Outpatient Oncology Setting: A Qualitative Study

**DOI:** 10.1177/08445621251396991

**Published:** 2025-11-24

**Authors:** Sarah Jane Quinn, Vera Caine, Olga Petrovskaya

**Affiliations:** 1 University of Victoria, Victoria, BC, Canada; 2 University of Alberta, Edmonton, AB, Canada

**Keywords:** patient portal, electronic health record, oncology, nursing, qualitative, actor-network theory

## Abstract

**Background:**

In November 2022, Alberta Health Services launched a new province-wide electronic health record, Connect Care (Epic), with a tethered patient portal, MyAHS Connect, across all Cancer Care Alberta sites. Oncology patients now can view their health record (including results), view and manage appointments, enter data directly into their chart, and securely message their health care team.

**Purpose:**

To explore how an online patient portal effects nurses and the health care team's work in an outpatient oncology setting.

**Methods:**

A descriptive qualitative method was used for this research study. 15 health care providers were recruited (12 registered nurses, 2 medical oncologists, 1 clerical worker). Data was analyzed using thematic analysis with a technology-in-practice sociomaterial theoretical perspective informing our approach.

**Results:**

Three main themes were generated: the invisibility of nurses’ responsibility of supporting patient portal use, access to the portal shapes a new type of patient, and MyAHS Connect is as good as the networks of care provision in which it is embedded.

**Conclusion:**

This qualitative study details how patient access to the portal changed the ways that health care providers are working but the degree of this change was highly influenced by patient use of the portal, staff's use of the electronic health record, and the greater system context. This research highlights the substantial role of nurses when patient portals are used in health care practice settings.

## Background and Purpose

Online patient portals are ever-increasing in uptake in health care internationally and in Canada, with oncology patients known to be one of the highest user populations of these systems (Suresh et al., 2025). Online portals allow patients to view their health information, test results, and treatment schedules, request that data be added or corrected in their chart and send and receive messages from their health care team from either a web browser or the mobile app version of the portal (AHS, 2024). People with cancer rate perceived benefits of using online portals higher than other patient populations accessing this technology (Rexhepi et al., 2020). In the Canadian province of Alberta (population approx. 4.7 million), its health authority, Alberta Health Services (AHS), implemented a clinical information system, Connect Care (Epic), from 2019 to 2024 (AHS, 2024), which includes a providers’ EHR and a patient portal, MyAHS Connect (AHS, 2024). The Epic system is widely utilized internationally (Epic, 2025) and in Canada (Digital Health Canada, 2024).

Alberta has a unique history of patient portals: there are two province-wide portals, both launched in 2019, (i) tethered MyAHS Connect launched in 2019–24 by AHS as part of Epic's Connect Care and (ii) view-only My Personal Records launched by the Government of Alberta's branch, Alberta Health, pulling information from a legacy EHR, Netcare (Government of Alberta, 2023, 2024). The history of these two Alberta's portals is well documented (Avdagovska, Stafinski, et al., 2020; Santos et al., 2021) and early experiences of patients and providers related to portal enrolment and use have been reported (Avdagovska, Ballermann, et al., 2020; Graham et al., 2020; Santos et al., 2021).

In November 2022, Connect Care launched across all oncology settings in Alberta (known as Cancer Care Alberta, CCA), enabling all patients visiting a provincial cancer centre to sign up for MyAHS Connect (MAC). Once patients sign up, they can instantly view their blood work, diagnostic imaging and pathology reports, and directly message their health care team. MAC also prompts patients to fill in their medication and health histories and complete their pre-visit questionnaires, MySymptom Report (MSR) up to 72 h prior to their follow up appointments.

Patient-provided data in MSRs is collectively referred to in organizational, quality-improvement, and research discourses as patient-reported outcome measures (PROMs). CCA has been collecting PROMs via pre-visit questionnaires since 2012 using a combination of the Edmonton Symptom Assessment System - revised (ESAS-r) and the Canadian Problem Checklist (Cuthbert et al., 2019), using paper forms prior to the launch of the online patient portal MAC. PROMs collection concurrently accomplishes the goal of improving patient assessments in the short-term, while also improving care long-term by supplying health data for research (Cuthbert et al., 2019). MSR tools are updated and modified over time (Watson et al., 2024).

With the launch of MAC, online-entered MSR patient data can now be captured for both telephone and in-person appointments with physicians. Moreover, when patients fill out their MSR online, the system generates additional questions for any symptoms rated four or higher. Patients can also select if they would like referrals to additional supportive services. No referrals are automatically sent via MSR, however staff review these requests during in-person clinic visits. Patients who do not fill out the online MSR via MAC are given a paper copy during their clinic visit. It is acknowledged that simply collecting PROMs does not improve patient outcomes, rather health care providers must know how to interpret these scores and then act on them accordingly (Snyder et al., 2019).

Published reviews of primary research focused on patient portals commonly report the actual or perceived patient benefits such as promoting self-management, patient centred-care, and improved health outcomes (Antonio et al., 2020; Benjamins et al., 2021; Brands et al., 2022; Carini et al., 2021; Coughlin et al., 2018; Damen et al., 2022; Risling et al., 2017; Suresh et al., 2025). These positive effects often stem from a patients’ convenient and timely access to their laboratory and imaging test results (Petrovskaya et al., 2023). Often, health care providers tend to speak more cautiously of patient portals, particularly in the oncology setting. Common concerns cited by health care providers include doubts about the accuracy of patient-entered information, increased patient anxiety, perceived increased workload, and lack of training for portal use (Petrovskaya et al., 2023). Oncologists express apprehension regarding patients viewing their abnormal or confusing results online prior to an in-person discussion (Lam et al., 2024; Suresh et al., 2025).

While patient portal research is rapidly growing, studies examining the contribution of nurses in supporting patients via EHRs and patient portals is a nascent field even though nurses play important roles in care coordination, in particular in oncology (Gerber et al., 2017; Laccetti et al., 2016; Stetiz et al., 2020). In a scoping review examining which nursing communication strategies increase patient engagement, patient portals were acknowledged to provide nurses with new communication methods to increase patient satisfaction (Crivelli et al., 2024). Shelley et al.'s (2024) systematic review found that health information systems in oncology settings increased communication between nurses and patients affecting nursing work of assessing, diagnosing, and planning. Shelley et al. (2024) observed that reviewed studies often left out the role nurses play in non-physical, holistic, patient needs. This review, however, was quite broad, including different information systems in both in-patient and out-patient settings such as health records, portals, remote symptom monitoring tools, and clinical decision-making tools.

It is projected that 1 in 2 Albertans will be diagnosed with cancer by 2050 (Alberta Health Services Cancer Strategic Clinical Network, 2022) so use of MAC in Alberta is only expected to increase. As of July 2025, less than three years after portal rollout, 66.5% of Cancer Care Alberta patients have an active MAC account (A. Wales, Cancer Care Alberta, personal communication, July 17, 2025). Yet, locally, patient portal's impact on care team communication such as electronic messaging, and effects of patient access to their test and imaging results in real-time, have not been studied in the oncology context in the province. Further, a province-wide EHR with the linked MAC patient portal is in existence in oncology settings only since November 2022. In addition to using other MAC functions, patients with cancer are now entering their symptoms (MSR or PROMs) pre-appointment into the portal rather than completing paper forms in the clinic. A few international (predominantly US) qualitative studies (Alpert et al., 2018, 2019; Sieck et al., 2017) focused on provider-patient communication via a portal mostly captured physicians’ perspective, thus obscuring nurses’ role in care coordination and communication. This research gap necessitated studying nurses’ work in the EHR- and patient portal-supported oncology setting in Alberta.

### Study Purpose

Our research was guided by the question: What are the effects of an online patient portal on the health care team's work in an oncology setting? The primary goal was to understand the experiences of nurses; however, based on the interdisciplinarity of cancer care it was important to also invite clerical staff and medical oncologists to participate.

## Methodology and Theoretical Approach

This qualitative descriptive study (Sandelowski, 2010) utilized a thematic analysis (Braun & Clarke, 2006) and was informed by a technology-in-practice sociomaterial theoretical perspective. According to this perspective, technology is neither all-knowing nor is it a simple tool (Timmermans & Berg, 2003). This perspective is rooted in actor-network theory (ANT; Latour, 1996; Law, 2007; Mol, 2010), and explores how an actor, human or non-human, acts in relation to other actors and their environment. ANT views technology as non-neutral, that is, inevitably altering the way humans behave, and invites a focus on how humans and technology are in relation with one another and in a specific context (Petrovskaya, 2023). Technology is neither unquestionably positive nor a negative force distracting health care workers from direct care (Petrovskaya, 2023; Santos et al., 2021). ANT informed analyses of nursing practice bring to light what nurses are actually doing rather than promulgating habitual views of nurses’ role (Wynn & Garwood-Cross, 2024). The application of ANT to this research helps foreground the presence of a new actor (the portal) and draws our attention to how it alters the behaviour of all actors who interact with it (health care workers, patients) within the organizational context.

### Setting and Sample

Fifteen health care providers (12 nurses, 1 clerk, and 2 medical oncologists) were recruited from July to October 2024 from the outpatient department solid-tumour systemic therapy clinics at an urban adult cancer centre in Alberta. Health care providers’ EHR, Connect Care, and a patient portal MyAHS Connect, launched in this setting in November 2022, replacing the previous EHR system, Aria. Prior to Connect Care, staff used Aria for scheduling and documentation and accessed Netcare separately from their web browser to view all test results. Aria did not have a tethered patient portal. At the time of this study 18 months following Connect Care launch, staff were still adapting to the new system augmenting the existing EHRs Netcare and Aria as needed.

In this setting, multidisciplinary health care teams follow up with patients who require active treatment and review all pathology, imaging, and blood work with them, typically during in person visits to adapt plans of care. Members of the interdisciplinary health care team include clerks, nurses (including licensed practical nurses, registered nurses, and nurse practitioners), and medical oncology physicians. Additional team members may be involved in patient care if patients are referred for their services, such as dietitian, rehabilitation, and psychosocial supports. Nurses support clinics by assessing patients and provide patient education on topics of chemotherapy, medications, nutrition, symptom management, and systemic navigation. Another group of nurses, trial nurses, support clinical research.

Nurses (*n* = 12) were recruited through a virtual information session and a recruitment email circulated to all nurses in the department. The study included nurses working in the outpatient department in various roles: nurses who see patients in clinic, nurses who support patients between clinic visits in telephone triage, nurses who recruit and support patients through clinical trials, and nurse coordinators who triage new oncology patient referrals and act as navigators for patients entering the system.

A recruitment email was also sent by a third party to all clerical staff and all medical oncologists at the centre; one clerical staff and two medical oncologists were recruited into the study. Clerical staff at the centre are responsible for various roles including scheduling, checking patients in for their in-person appointments, coordinating and scheduling patients’ tests/procedures, and working in the clinics as medical office assistants (rooming patients, taking height, weight, vital signs, and notifying nursing if patients have concerns requiring nursing assessment). Medical oncologists at the centre diagnose, treat, and support patients on systemic therapy cancer treatments including chemotherapy, targeted therapy, and immunotherapy.

### Protection of Human Subjects

Ethics approval was granted through the appropriate institutions. Operational approval was also obtained. After participants provided informed consent, interviews took place in a private setting. The primary researcher works in the setting where the study was conducted; however, they hold no authority over colleagues who agreed to participate in the study. Data management procedures adhered to ethical standards.

### Data Collection

Data was collected through semi-structured interviews from July to October 2024 either in person or on Zoom and ranged from 28–72 min (average 49 min). The primary author conducted all interviews and received training in conducting qualitative interviews as part of their graduate education. To maintain consistency during interviews, an interview guide (see supplemental material) was created and included questions such as, can you describe a typical situation when a patient comes to the clinic to see you, and this patient uses MyAHS Connect? Do you feel the presence of the MyAHS Connect patient portal changed your workflows? If yes, in what ways? Preliminary analysis occurred concurrently with data collection, which allowed adaptation of interview questions to more thoughtfully explore emerging themes. All interviews were recorded using a password protected audio recording device, stored on a secure shared drive, and transcribed using the Whisper.ai app which does not store any information in cloud technology. All study participants were emailed a $40 Amazon e-gift card following their interview to acknowledge their time contributed to the study. Data collection was stopped when no new insights were gained.

### Data Analysis

Thematic analysis (Braun & Clarke, 2006) was utilized to analyze data. The primary author first became familiar with the data by listening to the recorded interviews multiple times while reading the transcripts. Initial codes were created by the primary author by forming words or sentences to describe the main ideas in each section or subsection of a transcript. Following this, the entire research team, first individually then as a group, compared and integrated codes across interviews to form broader themes. Themes were then continuously refined with collaboration from all authors. This was an iterative process. Effort was taken to ensure themes had adequate interview data to support them and that each theme supported the research question. Our research team includes members with lived experience with cancer care and with extensive experience in patient portal research and technology-in-practice/actor-network theory; only the primary author works in the setting where the study was conducted.

Use of the technology-in-practice sociomaterial perspective enabled both data collection and analysis to focus on how the presence of the portal affects work processes and on how a non-human actor (i.e., EHR technology) can have agency versus focusing solely on human actors and their personal feelings or preferences regarding the portal. This theoretical approach also allowed for expansion of each theme to consider how socially complex health care system plays a role in human-technology interactions.

To uphold rigor in this study, we focused on credibility and transferability. The primary researcher was transparent and aware of their dual role (researcher and a nurse working at the study site). Credibility was ensured by selecting an appropriate sample size, actively listening during the interviews, using probing questions, and monitoring the accuracy of transcriptions. We provided contextual information about the clinic where the study was set and about patient portals in the province of Alberta, Canada (i.e., thick description); this will help the reader to establish similarities and differences with their specific contexts to determine the degree of transferability (as opposed to statistical generalizability) of the findings.

## Results

Fifteen participants with varying years of working experience were recruited including, 12 registered nurses, 1 clerk, and 2 medical oncologists (see Table 1 for participant demographics). Considering the research question of how the portal technology, MyAHS Connect (MAC), impacted health care provider work in the outpatient oncology setting, three key themes were identified: the invisibility of nurses’ responsibility of supporting patient portal use, access to the portal shapes a new type of patient, and MAC is as good as the care networks in which it is embedded (see Table 2 for an overview of the themes and subthemes).

**Table 1.** Participant Demographics.

Sample Characteristics	*n*
Gender	
Female	13
Male	2
Role	
Clerk	1
Nurse	12
Medical Oncologist	2
Age Range	
20-29 yrs old	2
30–39 yrs old	7
40–49 yrs old	1
50–59 yrs old	5

**Table 2.** Themes and Subthemes.

The invisibility of nurses’ responsibility of supporting patient portal use	
Managing in-basket messages from patients	
Managing electronic patient-entered data	
Access to the portal shapes a new type of patient	
Increased patient access to information alters social dynamics of care	
Increased access increases patient independence	
Increased access creates new considerations for patient anxiety and safety	
MyAHS Connect is as good as the networks of care provision in which it is embedded	
Health care provider support is critical for patients’ portal use	
Individualization of health care provider use of linked electronic health record	

### The Invisibility of Nurses’ Responsibility of Supporting Patient Portal use

#### Managing in-Basket Messages from Patients

Nurses in this study described being tasked with the significant responsibilities of handling the influx of work created by patient's ability to directly message staff via their patient portal. Physician staff are not responsible for checking and responding to these messages. After the launch of MAC, nursing staff realized that patients were utilizing this direct messaging feature, which presented a safety concern for nurses in regard to who would be addressing and triaging these messages. The department decided to create a new role for a nurse titled, “in-basket management.” Anytime a patient sends a message via the portal, the message gets filtered into one of two in-baskets in the EHR: “Patient Advice Request” or “Patient Scheduling Request.” The in-basket management nurse is responsible for reading, triaging and then responding to patient messages.

From a practice standpoint, nurses raised concerns about this role and the difficulty in both appeasing the patient and the physician simultaneously when the questions sent in were not something a nurse could address independently. “I think that in some instances, some nurses are being asked to potentially work outside of their scope of practice . . . So, a lot of things that are coming in, we can’t address. Yet we’re the ones assigned to address everything that comes in.” (Clinic Nurse #7)

It is not clearly indicated on the patient screen of MAC that patient's electronic messages will be read and triaged by nurses. It appears from the patient's end that they are messaging their physician directly. One nurse spoke to how this raises concerns for confidentiality, as patient messages are visible for the entire nursing department as opposed to their individual physician. Multiple nurses expressed how they have received complex questions via the in-basket, such as how to interpret results of radiology scans or plans for future treatment. Often nurses felt torn between appeasing the patient by reaching out to the physician or appeasing the physician by asking the patient to hold onto their concerns until the next clinic visit. One nurse spoke to their personal struggle in addressing in-basket questions from patients. “I find the ones that are more troublesome are when you have to message the doctor and then you have […] I feel like I get pushed back from the doctor I work with. And then I also don't want to be rude to the patient, but sometimes I will say, ‘Can you please write this down? We'll address this at your next visit’.” (Clinic Nurse #5)

Despite concerns with managing the influx of messages, nurses also spoke to ways that the in-basket messaging functionality positively impacted their work. Nurses expressed how being able to message one of their patients about a non-urgent concern saved time and is more convenient. The messaging feature also allows patients to send in photos to their health care team which makes it possible to engage in visual assessments. These photos are automatically uploaded to the patient's chart once sent in. Nurses can also send patients attachments and resources via MAC instantly. “I find what's really helpful is actually sharing resources with patients through it […] I can electronically send the information and they have that in hand when they feel that it's safer for them to kind of figure out what they want to pursue with regards to resources available to them.” (Clinic Nurse #1)

#### Managing Electronic Patient-Entered Data

In addition to messaging their health care team directly, patients can also enter their health history, request edits to their medication lists, and are prompted to enter their MySymptom Report (MSR) in advance of appointments. These MSR responses are then primarily, or only, used by nurses in the clinic setting. “But the majority of doctors I work with rely on nursing to use that data […] because they simply just don't have the time.” (Clinic Nurse #6). The medical oncologist (#2) pointed out, that “I'm assuming they [patients] will go through the MSR they report online or . .. on a paper. The nurses usually have that sheet … and then they just give us the report. So, do I see the MSR? Rarely. So, I just hear what the nurses are doing.”

Both medical oncologists also acknowledged that they are unsure where to find a patient's completed MSR if pre-entered online. Some nurses are now being asked by the physicians to review the MSRs for virtual (i.e., over the phone) patient follow-up visits. Previously, the only nursing role in virtual patient care was ensuring there were no orders entered by the physician that needed to be processed by nurses. “So, me and my doctor have an agreement that part of my duty for a virtual patient is to go and review the MSR, which … I don't think it would get reviewed if I didn’t […] I have to go and review the MSR after the fact. And if the doctor hasn’t mentioned anything in their note about these high-rated things, I’ll have to give them [the patient] a call and make sure they’re addressed.” (Clinic Nurse #6)

When patients enter their MSR via MAC, there are additional symptom assessment questions that are auto populated based on the scores of their symptoms. Nurses spoke very highly of this additional information and how much they valued it during their in-clinic assessments. It is a nurse's responsibility to enter all patient reported outcome measures (i.e., MSRs or PROMs) into the EHR; however, when patients have already done so online, there is no further work needed. It is important to note that nurses are only utilizing this information during clinic with patients and are not yet expected to routinely review PROMs in-between patient clinic visits. PROMs entered electronically do not replace in person or phone follow up.

Nurses in this study consistently reported that MAC linked to the EHR did not substantially decrease their workloads but instead altered the type of work they are doing. “Before … we were on Connect Care … we were filling out all the paperwork, … all the requisitions, … blood work, filling them, everything by handwriting it out… So [the Connect Care EHR] taken a lot of that out. But I do feel it's partially been replaced by this easy access … the patients have, where we're spending a lot more time reassuring or rebooking or changing things because they've seen something in their chart that needs to be fixed.” (Clinic Nurse #2)

### Access to the Portal Shapes a new Type of Patient

#### Increased Patient Access to Information Alters Social Dynamics of Care

Access, often described by staff as increased access, unfettered access or open access that the patients now have to their health care team via the portal, has changed the ways patients engage and thus how staff are working with them. Due to increased access, more patient care is being completed electronically, and happens in-between in-person follow-up appointments. This is because patients are seeing test results or information on their portal and are trying to have issues addressed immediately instead of waiting for their next visit. *“*[A] son called the phone line because of a hemoglobin that was - I want to say 79 - wondering if his mom needed a transfusion and … that call would never happen had they not known the result.” (Clinic Nurse #4)

Another change noted is a shift in the patient–health care provider dynamic. Patients are learning about results in real time, and as pointed out by a physician in the study - as patients are only looking for their own results versus staff who are looking for all their patients’ results, patients are reviewing their results often before staff has a chance to do so. Thus, staff have to learn how to respond to patients contacting them about concerns that staff are not yet aware of. This differs from the traditional health care provider-patient relationship where patients only find out about their results when they see their physician. This change in dynamics holds the possibility to negatively impact trust between patients and health care providers. “I'm now having the patient call me first to say*, ‘*Hey I saw this what are we gonna do about it?’ And I just have to have a conversation … like, “I've paged the doctor they're gonna get back to me when I get a plan I'll get back to you.” […] So I think it does add some workflow because instead of you know me seeing the result, paging the physician, the physician giving me instructions and me calling updating the patient on the plan, they're calling me first. I also don’t know if it necessarily makes me look good or on top of things or increases trust if I haven’t seen the results yet.” (Clinical Research Unit Nurse # 3)

#### Increased Access Increases Patient Independence

Not surprisingly, nurses noted that patients are more literate and knowledgeable about their cancer because they have information available to them to process and research on their own. “I remember just in the very beginning of Connect Care rollout people were stunned and upset. Like a staff … upset that the patients can have access so quickly without understanding the result, but it's become so commonplace in our population to be signed up for this app or services that I think it's just accepted now. And people are more literate.” (Clinic Nurse #4)

Almost every participant acknowledged that a patient's ability to see their schedules and appointments was beneficial for both patients and staff. Numerous staff reported they are addressing less phone calls from patients to clarify when their appointments or tests are. This seems to be particularly important for patients who are on chemotherapy or participating in a clinical trial as they have numerous appointments and tests to keep track of. “They would call us for everything including appointment times, ‘When is this happening? When's my CT?’ That has dramatically decreased with patients having the independence to be able to find that information for themselves.” (Clinical Research Unit Nurse #1). Additionally, staff can now ask patients to check their portal for updates as opposed to having to call patients to notify them. A clerk mentioned: “So, I said, ‘Okay I will let your nurse know… and then you can just check [the portal] maybe later today or tomorrow [to make sure the update was posted]’ … I always say if it's not changed for tomorrow, you can call me back and I will follow up again.” (Clerk)

The increased level of patient knowledge due to access to test results has also impacted patient-health care provider dynamics in the clinic setting. In some instances, staff felt patients having this knowledge prior to the visit was helpful for their visit preparation. “Sometimes you're bringing them into the room you sit them down and they're like, ‘Oh yeah I want to make sure I review my TSH [thyroid-stimulating hormone] with you.’ So, they already know their TSH is elevated they want to know why, and they want to talk about it. Which is something you would talk about anyways, but they're already prepared for it, which I think is great.” (Clinical Research Unit Nurse #1)

#### Increased Access Creates new Considerations for Patient Anxiety and Safety

In other instances, staff encountered scenarios where patient anxiety due to trying to interpret results alone at home meant staff where spending significant time with patients teaching them what the results truly mean and attempting to “talk them off a ledge” or “peel them off the ceiling.” Some nurses occasionally altered the timing of their in-clinic assessments to minimize patient anxiety related to the test results they have already seen. “But then you know you go in and they're crying, and they don't even really want to talk to you because they just want to talk to the doctor because they think everything is progressed and they're doing worse.” (Clinical Research Unit Nurse #3)

Interestingly, one nurse recognized how patient anxiety is also substantial if patients are waiting for results; this nurse was unsure what is worse – if a patient is waiting for results or seeing results that they do not understand. One of the medical oncologists in the study also spoke about how more detail-orientated patients were scrutinizing their reports even prior to MAC rollout. “Before the anxious person was getting print outs, again, scrutinizing them and coming back with questions, but again it's the same individuals who's getting it online and doing the same thing.” (Medical Oncologist #2) Having patients review them prior to the visit allowed patients to formulate their questions, as opposed to going home, re-reviewing the report, and then having to call in and return with more questions at a later date.

With increased access patients can now cancel their appointments without having to call in and provide a reason or request a cancellation. This workload of getting the tests and appointments rebooked can be challenging and is often done by nurses or clinic clerks. “Like this morning, I had a cancellation notification that the patient canceled a scan virtually […] And it's like whoa, now how is it going to get rebooked? Who's responsible? Who's rebooking it? I have no idea.” (Medical Oncologist #1)

Staff have also found numerous urgent symptom messages in the in-basket despite staff instructing patients to not use the portal for any urgent needs. “[Patient's direct message]: ‘Well today well my heart rate is being really bad and is there a medication to help me with this and can I have an ECG done?’ I was like okay; I can’t leave this one and go home.” (Clinic Nurse #4) There were differing opinions about a preferred method of communication, such as phoning about urgent concerns or sending an instant message. Some participants felt like a patient's ability to send a message led to increased reporting of symptoms due to the ease of communication. This can be both positive in that patients may be more likely to report concerns or challenging if the messages are not noticed in a timely manner.

Many staff expressed frustrations that patients seemed to be unaware of their health care team's workload. Often staff reported handling messages or questions from patients about things still being worked on. “So, we get a lot of messages of patients being like, ‘the chemo's not booked yet when is it going to be booked, what time is my chemo?’ So that's a common question that we get which takes up a lot of time as well responding back to patients reassuring them that we're working on it.” (Clinic Nurse #9*)* Interestingly, staff also spoke about ways to mitigate these types of tensions when it comes to managing patient expectations.

### MyAHS Connect is as Good as the Networks of Care Provision in Which it is Embedded

#### Staff Support is Critical for Patients’ Portal use

This study highlighted that many factors have effects on the usefulness of MAC for both patients and staff. Success of the system is heavily dependent on staff's ability to support portal use through encouraging and educating patients on how to use it and anticipating patients’ needs when reviewing test results via the portal. One physician spoke about how they provide education for patients with each test with regards to what they are anticipating seeing in the results. “I kind of have to go through with them and be like, what am I looking for? Right. And how am I interpreting the scan so that they're not freaking out when they get the results the week before, you know, I see it. […] Because like you’re empowering people, which is important, but you can’t empower people without the baseline knowledge they need to actually interpret the results. Then you’re just literally throwing people into a fire.” (Medical Oncologist #1)

Staff who are investing time to explain to patients what results may be and what next steps would be based on those results, reported it was time well spent. This was particularly significant for blood work results that were reported as abnormal, when it may still be considered within the safe range for patients to receive chemotherapy, such as absolute neutrophil count and red blood cell counts.

Staff also reported creative ways patients use to clarify information they accessed on their portal including using Google or ChatGPT to interpret their radiology and pathology reports. Some nurses also reported that some patients are becoming self-aware of how viewing information via their portal makes them feel and choose to stop reviewing their results. “I've had a lot of patients that start off looking at their scans and then they stop because they don't understand fully what is being told. And then they say it stresses them out more rather than just going through it with the doctor.” (Clinic Nurse #3)

Staff, particularly nurses, spoke about how they are receiving more complex questions from patients regarding their care and thus are now having to speak in more detail about the results such as blood work and imaging with patients. One nurse no longer just tells patients that their blood work is good or fit to proceed with chemotherapy, but instead now describes why that is specific to the numbers. Nurses feel they still need to call patients to explain test results. “So I'll phone them just to say, “yahoo, you know we got another good result,” and but you know many patients have said to me even though they did get in and they looked, they say to me, ‘But I'm really grateful you called, because I don't always understand what they're saying.’ And I hear that quite a bit too actually. Which again reinforces to me that you should phone.” (Clinic Nurse #8)

#### Individualization of Health Care Provider use of Linked Electronic Health Record

We also found in this study that not only are patients utilizing the portal in very individualized ways, but staff's use of the EHR is individualized. This created some staff concerns with care potentially being inconsistent for patients depending on staff's willingness to learn new technology and incorporate it into their practice. The clerk shared how their fears of being audited or potentially doing something wrong in a patient's chart holds them back from exploring the functionalities of the EHR. “I don’t touch things I'm not familiar with because I'm scared, maybe I will touch it and then I'm gonna get audited or whatever… I only use it what my job is using, I don’t press anything that's oh, I didn’t know what that was.” (Clerk)

The study participants discussed differences among staff's use of MAC functionalities and the tendency to align with the physician's preferred way to use the technology. For example, one physician in the study is comfortable securely messaging patients on MAC but the other physician chooses to not communicate with patients via the portal. These preferences significantly alter how a nurse would address a patient's concern: forwarding it directly to the physician versus contacting the physician for an answer and then contacting the patient to relay that information. The clerk in the study also spoke to how each clinic within the cancer centre operates differently from the others, and the clerk's tendency is to favour the preference of the nurse they are working with. The clerk spoke specifically to how they do not review patient symptom reports (MSRs) in the clinic with patients. “No, and they [nurses] don’t want me to do that… It's my nurses’ choice… I think as what I’ve heard from other clerks every clinic is different… but it doesn’t apply to my clinic that I need to do that symptoms thing… I’ve heard from other clerks who work there that yeah, they do things different from what I do.” (Clerk).

Staff are feeling unsupported in their education regarding the patient portal. All staff in this study reported never having seen what the patient portal looks like unless they had their own personal account to access it, making patient education about the portal very difficult. “That's probably the biggest negative right now… that gap between what we see and what patients see.” (Medical Oncologist #1). Staff also described that patients were not prepared for this new technology; they only received sign-up information once the portal launched. Despite this, participants detailed how they worked together with patients to troubleshoot the portal. One nurse described how they were unsure how to teach patients to send in photos via MAC, so they practiced with a patient during a clinic while they were waiting to see their oncologist. “I had some downtime in clinic and I said [to a patient], ‘Can we practice? Can we try and upload a photo?’ So, I sat with her, so then I found we were able to upload it”. (Clinic Nurse #5) Overall, it was evident that staff felt strongly that their roles were still highly valued in a context where a patient portal is present and actively used by patients.

## Discussion

The aim of this study was to identify how the work of nurses and other health care providers (clerical staff and medical oncologists) has changed since the implementation of a patient portal in the large urban outpatient oncology centre in Alberta. Within the first two years post-implementation, the portal has changed when and how work is being completed by nurses, oncologists, and clerks involved in conducting clinic visits as well as the interpersonal dynamics between all health care team members. The impact of the portal was highly circumstantial, in that it was dependent on the individual use (patients, health care provider) of the portal on either end of the system. Participants were honest about how the portal can be a helpful tool to give patients more independence and to ease communication. However, it also requires mindful and intentional work to anticipate and mitigate its limitations. Ultimately, the power of the portal to impact care practices and health care providers’ work was highly dependent on how the interviewed providers interacted with it within the parameters set by the clinic's management. In other words, the management could make decisions about what functions of the portal will or will not be used. This aligns with the actor-network theory argument about a non-deterministic and non-essentialist nature of technology; what the portal in the clinic “is” does not equal to its technical design but rather becomes a product of political, economic, and other effects shaping care provision.

Laukka et al. (2020), in a qualitative systematic review examining health care providers’ experiences interacting with patients via electronic portal messaging, found that the messaging could have both positive and negative impacts and affected the work of various providers differently. For example, oncologists viewed increased communication via a portal as beneficial for patients (Alpert et al., 2019), but oncology nurses recognized that it increased the frequency of interactions and the workload (Gerber et al., 2017). Additionally, work can be completed more efficiently with the portal since messaging a patient is faster than phone calls and interactions are immediately documented into the patients record (Bishop et al., 2013). However, messaging requires specialized knowledge within a professional's scope to answer patient questions, and non-physician professionals had to ask for support from other appropriate providers to respond (Das et al., 2015). Our findings support observations from the above studies.

Our study found that nurses were assigned most of the new work at the intersection of the patient portal and provider portal (EHR). Participants described that patients were not aware that nurses triage and respond to their messages, as the portal is set up in a way that gives patients the impression they are messaging their oncologist directly. Nurses’ work of supporting patient care mediated by portal technology is invisible to the public. Allen (2015) argues that the majority of nursing work happens outside of direct patient encounters (i.e., invisible to the public, other health care providers, and policymakers) and encompasses care management elements crucial for patient care within a fragmented health care system.

Our study surfaces the emergence of a new nursing role, an in-basket manager; the role is important to support patient care yet is invisible. In line with actor-network theory, our study presents an empirical example of the effects new technology can have for the nursing workforce. In addition to clinic nurses conducting face-to-face patient visits and addressing patient concerns over the phone, a nurse in this new role is now spending an entire working day solely addressing electronic patient messages. This work requires a refined clinical and organizational knowledge to be able to appropriately handle patient queries and coordinate care with a minimal disruption of other providers’ work and the most benefit for the patient.

The introduction of a MyAHS Connect (MAC) patient portal has brought forward how nurses mainly take on the responsibility of reviewing and acting on PROMs. This was especially evident in the case of the scheduled telephone follow-up visits. Prior to MAC, nurses were not involved in addressing patient symptom concerns for such visits. Post-MAC implementation, some oncologists have asked their nursing team to review those patients’ MySymptom Reports (MSR or PROMs) in MAC as a safeguard to ensure all patient symptoms were addressed in case patients reported symptoms online but did not verbalize them over the phone. This is another example of how MAC has altered the nursing workflow as some nurses were now comparing MSRs questionnaires with oncologist's documentation and calling patients to complete additional assessments if necessary. This workflow, however, was not standardized throughout the department.

Although PROMs are collected in cancer care organizations across Canada (Canadian Partnership Against Cancer, 2018) and the interventional studies on PROMs are abundant (Balitsky et al., 2024), to the best of our knowledge, there is a dearth of empirical studies comparing, the actual role of nurses and physicians in acting on PROMs and the nurses’ work involved. In a breast and colorectal cancer clinic in Australia that utilizes PROMs, Girgis et al. (2009) compared the management of PROMs by oncology nurse telephone case workers versus medical doctors (general practitioners and oncologists) and found that more referrals for psychosocial resources were made by nurses. Basch et al. (2016), observed that at a US cancer centre, system alerts of patient-entered symptom concerns were emailed to the nursing team to follow up on and address between clinic visits. This echoes our findings that nurses play a large role in collecting PROMs and addressing symptoms reported by patients. This illustrates what Allen (2015) describes as nurses’ dual orientation toward the individual patients and the organizational priorities as PROMs are important organizational performance metrics taken to the next level by online patient portals.

Other studies in oncology settings with newly implemented PROMs reported that despite an expectation that nurses should act upon patient-reported symptoms, nurses were struggling to integrate PROMs into their workflow due to a lack of organizational strategies to do so (Thestrup Hansen et al., 2021) or felt that addressing PROMs sometimes required exceeding their scope of practice (Kotronoulas et al., 2017). Our study thus highlights an aspect of nurses’ work less frequently captured in the literature where nurses are the primary member of the health care team reviewing and acting on electronically entered PROMs, yet this work too seems invisible within the organizations.

A concept evident throughout each theme was team dynamics and the role that power plays within the health care team. Our discussion of power is informed by Michel Foucault's (1983) view of power as existing in interstices (i.e., being present in all interactions) and as not necessarily a force that oppresses and negates; power also entices people to act and shapes subjectivities. On the other hand, this conception of power should not overshadow the reality of health care with an unequal distribution of knowledge and expertise and different positions occupied by individuals. As much as nurses strive to be patient-centred and mitigate power inequalities in nurse-patient relationships, patient access to the portal resurfaced power dynamics and precipitated shifts in social relations.

In line with actor-network theory's view of agency arising within, and dependent upon, socio-technical networks, and Foucault's view that subjectivities are produced within the capillaries of power relations, one of our key themes was the emergence of a new kind of patient who is an active portal user. Foucault argued that subjectivities (i.e., what persons feel they are) are not inherent but rather emerge and are shaped within relationships among people, institutions, and discourses in everyday life. In this way, “patients” are not pregiven within the healthcare system, but rather the “kinds of patients” (e.g., a proactive and literate patient or, in contrast, a patient with lower health literacy or less tools and knowledge on technology use) are emerging depending on the context. Patients accessing portal information exhibited what participants in the study described as increased autonomy. Staff observed that patients seemed “smarter” and better informed as they studied their pathology and radiology reports at home, researched details on the internet, or contacted the clinic immediately to clarify results. Patients’ access to their information meant they often knew more about their test results in that moment-in-time than their care providers.

Despite patients’ increased confidence, staff we interviewed felt that patients were still highly dependent on their care team to interpret test results and answer their questions. Published research overwhelmingly supports these findings highlighting that most patients accessing test results online desire further explanations (Santos et al., 2021). Relatedly, patients’ anxiety is often highly dependent on how quickly they can discuss their test results with their health care provider (Petrovskaya et al., 2023). However, Alpert et al. (2019) found that all 13 oncologists in their study expressed concerns that their workloads would not allow them to meet patient expectations for rapid communication following immediate test result release.

Patient portals have the potential to affect nurses’ autonomy in the workplace. Nurses described how the portal created challenges for them to work within their scope of practice. A nurse's sense of autonomy is fostered by completing tasks according to their own judgement and is known to increase their sense of meaning in their work (Both-Nwabuwe et al., 2020). In our study, the position of nurses in relation to patients and the portal sometimes made it difficult for them to continue to work autonomously. Nurses described their role as gatekeepers to the oncologists they work with. Allen (2015) similarly describes nurses as the mandatory health care providers present at different care interfaces throughout a patient's care trajectory. Nearly all nurses in the study described being in scenarios where they had to consider contacting oncologists regarding patient concerns between clinic visits - scenarios created by patients’ ability to message via the portal at any time. Both-Nwabuwe et al. (2020) argue that professional autonomy, the ability to carry out work without requiring constant permission from others, contributes to meaningful nursing work. This perspective offers a potential explanation as to why nurses struggle with in-basket management; a role that provides little professional autonomy in a context where patients think they are messaging their physician.

Our findings illuminated significant differences in how staff members (even within their respective professional groups) engage with the patient portal such as responding to patients’ messages. Similarly, Gerber et al. (2017) observed differences in preferences for communication styles among oncologists. This raises an interesting question for health care settings implementing patient portals and EHRs that allow for substantial options. Increased options means that care processes are less standardized as providers and patients find their own preferred method to carry out their work and interactions. Mannion and Exworthy (2017) explored how standardization and customization are competing logics in health care and argued that it is critical that health care research focuses on these tensions.

Systematic reviews of published research on patient portals (Antonio et al., 2020; Petrovskaya et al., 2023) observed variable and inconsistent impact of patient portal technology on health care providers’ workload and called for contextualized studies examining this topic. In our study, staff strongly foregrounded the topic of the patient portal's effect on their work efficiency and workload. It was clear that productivity, efficiency, and effective care management, as organizational priorities, are taken seriously. Nurses categorized patient portal functionalities and use patterns as either positive or negative based on whether the portal allowed to save time or, in contrast, increased workload. Nurses, however, did not succumb to the efficiency discourses; their foregrounding of effective care management still centered their professional aspiration of patient-centred care.

We argue that the success of the portal is not predetermined and fixed but rather depends on the socio-technical networks in which it is embedded. In our study, staff explained that they and patients relied heavily on one another for support on how to use the portal, noticing the lack of educational support within the organization. In our study, staff felt uncertain about some portal features and appropriate types of portal interactions with patients. Equally, nurses felt ill-prepared to support patients on how to enroll into and use the portal. Nurses shared their concern about potential adverse outcomes related to patients’ unfamiliarity with the best and appropriate way to use the portal (e.g., canceling appointments, using portal messaging feature to send in urgent symptom concerns).

Nevertheless, over the nearly two years since the implementation of the portal, this technology required substantial changes in the context of patient education in the clinic: nurses now invest time in teaching patients how to utilize the portal while oncologists teach them how to interpret test results. Nurses and oncologists considered such anticipatory (in contrast to reactive) teaching a worthwhile investment of time. Allen (2015) highlighted nurses’ critical role in identifying barriers to patients’ smooth progression through the health care system and how they work to mitigate these barriers. Numerous participants in our study viewed the study as an opportunity to voice their ideas for improving the portal and EHR to benefit care delivery.

### Recommendations

All participant groups (nursing, clerical, medical oncologists) expressed a need for more education around the portal both for themselves and for the patients to maximize its benefits. Most noticeably, staff urged for the ability to be able to view the patient portal without having to create their own personal account. Many participants felt never having seen the portal created a major gap in caring for patients utilizing this technology. Step-by-step instruction sheets for patients on how to send in photos and documents will be important. Many participants also urged for practical changes to the portal features such as eliminating the appointment cancelation feature (instead suggesting patients can request cancelations to be reviewed and approved by staff) and making it more obvious on the patient portal that their messages will be read and addressed by multiple nurses in the department. The medical oncologists suggested having patients’ pre-entered MSRs (PROMs) pop up for them to review automatically when they initiate a patient visit in the EHR so they can review this data without having to search for it in the chart. Nurses recommended more support and direction for the in-basket management role. This is not surprising considering that this role requires frequently consulting other team members as patient messages often require either a physician's scope of practice or a depth of understanding of one clinic's daily operations that not every nurse in the department would be familiar with. Some participants also spoke fondly how the proxy-access feature (ability for MAC users to allow a proxy person access to their portal) allowed certain patient populations more equitable access to the portal. This was especially useful for patients who do not speak English, have disabilities, or are not comfortable with portal technology. Thus, we would also recommend portal technology with a proxy-access feature available when feasible.

For other sites implementing patient portals, nurses’ capacity and roles need to be considered, as we have found nurses were assigned additional workloads created by portals. At the site of this study, an additional 1–2 nurses were required to be staffed daily to take on the workload of triaging and respond to patients’ in-basket messages. It would be interesting for future portal research to focus on how nurses in other oncology care settings are managing the work created by patient portals. We recommend that portals are viewed as a supportive technology in health care, but not as a replacement of health care providers. We also recommend that health care providers take the time to explore their patients’ portal behaviour to see if portal use could be optimized with more education and support. It is important that portal use is discussed with patients from early contact with the cancer centre and that patients’ needs related to portal use, including how to utilize it and how to interpret the information on the portal, especially test results, are anticipated to mitigate any potential negative effects.

### Strengths and Limitations

This study contributes to the body of knowledge on how patient portals transform oncology nurses’ work in Canada. Our theoretical framework, technology-in-practice sociomaterial perspective and actor-network theory, offers a unique perspective on how organizational context plays a role in portal use and the effects of a new non-human actor (MAC) on nurses’ work. The focus of this study was on nurses. Thus, the small number of clerks (*n* = 1) and oncologists (*n* = 2), although representative of the so-called social activity system (Allen, 2015) in this clinic, should be taken into consideration when transferring our findings to other cases. We did not engage in member checking; we instead relied on the first author's familiarity and experiences within the setting.

## Conclusion

Patient portals in the oncology setting alter the work of health care providers. The degree of this change is highly dependent on the patient use of the portal, the health care provider's use of the EHR, and the organizational context. Nurses play a significant role in supporting portal use by patients, particularly in managing patient messages and patient data sent in through the system. However, this work remains largely invisible to patients, the public, and other members of the health care team. Patients’ knowledge of their cancer and care is ever-increasing with portal use meaning that health care providers have to adapt their work and relationships with patients in recognition of this. Successful portal use requires organizational support of health care providers so that they can proactively support patient portal to prevent negative outcomes. Patient portals do not replace health care team's support such as interpretation of portal information. Ultimately, health care providers (clerical staff, nurses, and medical oncologists) remained highly valued in a portal context. The collaborative nature of oncology was highlighted by the portal as health care providers and patients worked together to navigate this technology and improve patient-centered care.

## Supplemental Material

sj-docx-1-cjn-10.1177_08445621251396991 - Supplemental material for The Effects of an Online Patient Portal on Nurses’ and the Health Care Team's Work in an Outpatient Oncology Setting: A Qualitative Study Supplemental material, sj-docx-1-cjn-10.1177_08445621251396991 for The Effects of an Online Patient Portal on Nurses’ and the Health Care Team's Work in an Outpatient Oncology Setting: A Qualitative Study by Sarah Jane Quinn, Vera Caine and Olga Petrovskaya in Canadian Journal of Nursing Research

## References

[bibr1-08445621251396991] Alberta Health Services. (2024). What you can do with MyAHS Connect. https://www.albertahealthservices.ca/assets/info/cis/if-cis-cc-mac-features.pdf

[bibr2-08445621251396991] Alberta Health Services Cancer Strategic Clinical Network. (2022). Future of cancer impact in Alberta- Summary. https://www.albertahealthservices.ca/assets/about/scn/ahs-scn-cancer-future-of-cancer-impact-summary.pdf

[bibr3-08445621251396991] Allen D. (2015). The invisible work of nurses: Hospitals, organisation and healthcare. Routledge.

[bibr4-08445621251396991] Alpert J. M. Morris B. B. Thomson M. D. Matin K. Brown R. F. (2018). Implications of patient portal transparency in oncology: Qualitative interview study on the experiences of patients, oncologists, and medical informaticists. JMIR Cancer, 4(1), e5. 10.2196/cancer.8993 PMC5891668 29581090

[bibr5-08445621251396991] Alpert J. M. Morris B. B. Thomson M. D. Matin K. Brown R. F. (2019). Identifying how patient portals impact communication in oncology. Health Communication, 34(12), 1395–1403. 10.1080/10410236.2018.1493418 29979886 PMC6320725

[bibr6-08445621251396991] Antonio M. G. Petrovskaya O. Lau F. (2020). The state of evidence in patient portals: Umbrella review. Journal of Medical Internet Research, 22(11), e23851. 10.2196/23851 PMC7688386 33174851

[bibr7-08445621251396991] Avdagovska M. Ballermann M. Olson K. Graham T. Menon D. Stafinski T. (2020b). Patient portal implementation and uptake: Qualitative comparative case study. Journal of Medical Internet Research, 22(7), e18973–e18973. 10.2196/18973 PMC7427986 32716308

[bibr8-08445621251396991] Avdagovska M. Stafinski T. Ballermann M. Menon D. Olson K. Paul P. (2020). Tracing the decisions that shaped the development of MyChart, an electronic patient portal in Alberta, Canada: Historical research study. Journal of Medical Internet Research, 22(5), e17505. 10.2196/17505 PMC7284487 32452811

[bibr9-08445621251396991] Balitsky A. K. Rayner D. Britto J. Lionel A. C. Ginsberg L. Cho W. Wilfred A. M. Sardar H. Cantor N. Mian H. Levine M. N. Guyatt G. H. (2024). Patient-reported outcome measures in cancer care an updated systematic review and meta-analysis. JAMA Network Open, 7(8), e2424793. 10.1001/jamanetworkopen.2024.24793 PMC11322847 39136947

[bibr10-08445621251396991] Basch E. Deal A. M. Kris M. G. Scher H. I. Hudis C. A. Sabbatini P. Rogak L. Bennett A. V. Dueck A. C. Atkinson T. M. Chou J. F. Dulko D. Sit L. Barz A. Novotny P. Fruscione M. Sloan J. A. Schrag D. (2016). Symptom monitoring with patient-reported outcomes during routine cancer treatment: A randomized controlled trial. Journal of Clinical Oncology, 34(6), 557–565. 10.1200/JCO.2015.63.0830 26644527 PMC4872028

[bibr11-08445621251396991] Benjamins J. Haveman-Nies A. Gunnink M. Goudkuil A. De Vet E. (2021). How the use of a patient-accessible health record contributes to patient-centered care: Scoping review. Journal of Medical Internet Research, 23(1), e17655. 10.2196/17655 PMC7834934 33427683

[bibr12-08445621251396991] Bishop T. F. Press M. J. Mendelsohn J. L. Casalino L. P. (2013). Electronic communication improves access, but barriers to its widespread adoption remain. Health Affairs, 32(8), 1361–1367. 10.1377/hlthaff.2012.1151 23918479 PMC3817043

[bibr13-08445621251396991] Both-Nwabuwe J. M. C. Lips-Wiersma M. Dijkstra M. T. M. Beersma B. (2020). Understanding the autonomy–meaningful work relationship in nursing: A theoretical framework. Nursing Outlook, 68(1), 104–113. 10.1016/j.outlook.2019.05.008 31427079

[bibr14-08445621251396991] Brands M. R. Gouw S. C. Beestrum M. Cronin R. M. Fijnvandraat K. Badawy S. M. (2022). Patient-centered digital health records and their effects on health outcomes: Systematic review. Journal of Medical Internet Research, 24(12), e43086. 10.2196/43086 PMC9816956 36548034

[bibr15-08445621251396991] Braun V. Clarke V. (2006). Using thematic analysis in psychology. Qualitative Research in Psychology, 3(2), 77–101. 10.1191/1478088706qp063oa

[bibr16-08445621251396991] Canadian Partnership Against Cancer. (2018). 2018 cancer system performance report. https://s22457.pcdn.co/wp-content/uploads/2019/01/2018-Cancer-System-Performance-Report-EN.pdf

[bibr17-08445621251396991] Carini E. Villani L. Pezzullo A. M. Gentili A. Barbara A. Ricciardi W. Boccia S. (2021). The impact of digital patient portals on health outcomes, system efficiency, and patient attitudes: Updated systematic literature review. Journal of Medical Internet Research, 23(9), e26189. 10.2196/26189 PMC8459217 34494966

[bibr18-08445621251396991] Coughlin S. S. Caplan L. Young L. (2018). A review of web portal use by oncology patients. Journal of Cancer Treatment and Diagnosis, 2(6), 1–6. 10.29245/2578-2967/2018/6.1154 30680374 PMC6342494

[bibr19-08445621251396991] Crivelli A. F. Barello S. Acampora M. Bonetti L. (2024). Uncovering nursing communication strategies and relational styles to foster patient engagement in oncology: A scoping review. Healthcare, 12(13), 1261. 10.3390/healthcare12131261 38998795 PMC11241268

[bibr20-08445621251396991] Cuthbert C. A. Watson L. Xu Y. Boyne D. J. Hemmelgarn B. R. Cheung W. Y. (2019). Patient-reported outcomes in Alberta: Rationale, scope, and design of a database initiative. Current Oncology, 26(4), e503–e509. 10.3747/co.26.4919 PMC6726254 31548818

[bibr21-08445621251396991] Damen D. J. Schoonman G. G. Maat B. Habibović M. Krahmer E. Pauws S. (2022). Patients managing their medical data in personal electronic health records: Scoping review. Journal of Medical Internet Research, 24(12), e37783. 10.2196/37783 PMC9832357 36574275

[bibr22-08445621251396991] Das A. Faxvaag A. Svanæs D. (2015). The impact of an ehealth portal on health care professionals’ interaction with patients: Qualitative study. Journal of Medical Internet Research, 17(11), e267–e267. 10.2196/jmir.4950 PMC4704899 26601678

[bibr23-08445621251396991] Digital Health Canada. (2024). Epic collaborative: Epic collaborative update. https://digitalhealthcanada.com/wp-content/uploads/2024/03/Digital-Health-Canada-Epic-Collaborative-Update-Mar-2024_v2.pdf

[bibr24-08445621251396991] Epic. (2025, January). Our story in a nutshell. https://www.epic.com/about/

[bibr25-08445621251396991] Foucault M. (1983). The subject and power. In Dreyfus H. L. Rabinow P. Foucault M. (Eds.), Michel foucault, beyond structuralism and hermeneutics (2nd ed., pp. 208–226). University of Chicago Press.

[bibr26-08445621251396991] Gerber D. E. Beg M. S. Duncan T. Gill M. Craddock Lee S. J. (2017). Oncology nursing perceptions of patient electronic portal use: A qualitative analysis. Oncology Nursing Forum, 44(2), 165–170. 10.1188/17.ONF.165-170 28222081 PMC7066859

[bibr27-08445621251396991] Girgis A. Breen S. Stacey F. Lecathelinais C. (2009). Impact of two supportive care interventions on anxiety, depression, quality of life, and unmet needs in patients with nonlocalized breast and colorectal cancers. Journal of Clinical Oncology, 27(36), 6180–6190. 10.1200/JCO.2009.22.8718 19917842

[bibr28-08445621251396991] Government of Alberta. (2023). What is MyHealth Records? https://www.albertanetcare.ca/Projects/MyHealthRecords.htm#:∼:text=How20Does20MyHealth20Records20work,store20other20personal20health20information

[bibr29-08445621251396991] Government of Alberta. (2024, January). Alberta netcare EHR: Facts and stats section page. https://www.albertanetcare.ca/facts.htm#:∼:text=Alberta20Netcare20was20created20in,professionals2C20and20health20information%20regulators

[bibr30-08445621251396991] Graham T. A. D. Ali S. Avdagovska M. Ballermann M. (2020). Effects of a web-based patient portal on patient satisfaction and missed appointment rates: Survey study. Journal of Medical Internet Research, 22(5), e17955. 10.2196/17955 PMC7267992 32427109

[bibr31-08445621251396991] Kotronoulas G. Papadopoulou C. MacNicol L. Simpson M. Maguire R. (2017). Feasibility and acceptability of the use of patient-reported outcome measures (PROMs) in the delivery of nurse-led supportive care to people with colorectal cancer. European Journal of Oncology Nursing, 29, 115–124. 10.1016/j.ejon.2017.06.002 28720258

[bibr32-08445621251396991] Laccetti A. Chen B. Cai J. Gates S. Xie Y. Lee S. J. C. Gerber D. E. (2016). Increase in cancer centre staff effort related to electronic patient portal use. Journal of Oncology Practice, 12(12), e981–e990. https://ascopubs.org/doi/10.1200/JOP.2016.011817 https://doi.org/10.1200/JOP.2016.011817 10.1200/JOP.2016.011817 PMC5455586 27601511

[bibr33-08445621251396991] Lam K. Leung C. H. Soomro Z. Wathoo C. Weathers S. P. (2024). Perspectives from neuro-oncology providers on patient access to electronic health records: A survey study. CNS Oncology, 13(1), 1–5. 10.1080/20450907.2024.2352414 PMC11152585 38869443

[bibr34-08445621251396991] Latour B. (1996). On actor-network theory: A few clarifications. Soziale Welt, 47(4), 369–381. https://www.jstor.org/stable/40878163 .

[bibr35-08445621251396991] Laukka E. Huhtakangas M. Heponiemi T. Kujala S. Kaihlanen A.-M. Gluschkoff K. Kanste O. (2020). Health care professionals’ experiences of patient-professional communication over patient portals: Systematic review of qualitative studies. Journal of Medical Internet Research, 22(12), e21623. 10.2196/21623 PMC7755533 33289674

[bibr36-08445621251396991] Law J. (2007). Actor network theory and material semiotics. http://www.heterogeneities.net/publications/Law2007ANTandMaterialSemiotics.pdf

[bibr37-08445621251396991] Mannion R. Exworthy M. (2017). (Re) making the procrustean bed? Standardization and customization as competing logics in healthcare. International Journal of Health Policy and Management, 6(6), 301–304. 10.15171/ijhpm.2017.35 28812821 PMC5458790

[bibr38-08445621251396991] Mol A. (2010). Actor-network theory: Sensitive terms and enduring tensions. Kölner Zeitschrift für Soziologie und Sozialpsychologie. Sonderheft, 50(1), 253–269. https://hdl.handle.net/11245/1.330874

[bibr39-08445621251396991] Petrovskaya O. (2023). Technology and nursing. In Liscomb M. (Ed.), Routledge handbook of philosophy and nursing (pp. 481–493). Routledge.

[bibr40-08445621251396991] Petrovskaya O. Karpman A. Schilling J. Singh S. Wegren L. Caine V. Kusi-Appiah E. Green W. (2023). Patient and health care provider perspectives on patient access to test results via web portals: Scoping review. Journal of Medical Internet Research, 25, e43765. 10.2196/43765 PMC10623227 37856174

[bibr41-08445621251396991] Rexhepi H. Moll J. Huvila I. (2020). Online electronic healthcare records: Comparing the views of cancer patients and others. Health Informatics Journal, 26(4), 2915–2929. 10.1177/1460458220944727 32914701

[bibr42-08445621251396991] Risling T. Martinez J. Young J. Thorp-Froslie N. (2017). Evaluating patient empowerment in association with ehealth technology: Scoping review. Journal of Medical Internet Research, 19(9), e329. 10.2196/jmir.7809 PMC5640823 28963090

[bibr43-08445621251396991] Sandelowski M. (2010). What’s in a name? Qualitative description revisited. Research in Nursing & Health, 33(1), 77–84. 10.1002/nur.20362 20014004

[bibr44-08445621251396991] Santos A. D. Caine V. Robson P. J. Watson L. Easaw J. C. Petrovskaya O. (2021). Oncology patients’ experiences with novel electronic patient portals to support care and treatment: Qualitative study with early users and nonusers of portals in Alberta, Canada. JMIR Cancer, 7(4), e32609. 10.2196/32609 PMC8663539 34822338

[bibr45-08445621251396991] Shelley D. Davis D. Bail K. Heland R. Paterson C. (2024). Oncology nurses’ experiences of using health information systems in the delivery of cancer care in a range of care settings: A systematic integrative review. Seminars in Oncology Nursing, 40(2), 151579. 10.1016/j.soncn.2023.151579 38402020

[bibr46-08445621251396991] Sieck C. J. Hefner J. L. Schnierle J. Florian H. Agarwal A. Rundell K. McAlearney A. S. (2017). The rules of engagement: Perspectives on secure messaging from experienced ambulatory patient portal users. JMIR Medical Informatics, 5(3), e13. 10.2196/medinform.7516 PMC5516097 28676467

[bibr47-08445621251396991] Snyder C. Brundage M. Rivera Y. Wu A. (2019). A PRO-cision medicine methods toolkit to address the challenges of personalizing cancer care using patient-reported outcomes: Introduction to the supplement. Medical Care, 57(5), S1–S7. 10.1097/MLR.0000000000001089 PMC7400766 30985589

[bibr48-08445621251396991] Stetiz B. D. Unerthl K. M. Levy M. A. (2020). Characterizing communication patterns among members of the clinical care team to deliver breast cancer treatment. Journal of the American Medical Informatics Association, 27(2), 236–243. 10.1093/jamia/ocz151 31682267 PMC7647266

[bibr49-08445621251396991] Suresh U. Ancker J. Salmi L. Diamond L. Rosenbloom T. Steitz B. (2025). Advancing cancer care through digital access in the USA: A state-of-the-art review of patient portals in oncology. BMJ Oncology, 4(1), e000432. 10.1136/bmjonc-2024-000432 PMC11883497 40052188

[bibr50-08445621251396991] Thestrup Hansen S. Kjerholt M. Friis Christensen S. Brodersen J. Hølge-Hazelton B. (2021). Nurses’ experiences when introducing patient-reported outcome measures in an outpatient clinic: An interpretive description study. Cancer Nursing, 44(2), E108–E120. 10.1097/NCC.0000000000000808 32217877

[bibr51-08445621251396991] Timmermans S. Berg M. (2003). The practice of medical technology. Sociology of Health & Illness, 25(3), 97–114. 10.1111/1467-9566.00342 14498932

[bibr52-08445621251396991] Watson L. Link C. Qi S. Delure A. Chmielewski L. Hildebrand A. Barbera L. (2024). Designing and validating a comprehensive patient-reported outcomes measure for ambulatory cancer settings: The revised Edmonton symptom assessment system for cancer. JCO Oncology Practice, 20(12), 1764–1775. 10.1200/OP.24.00088 38954778 PMC11649173

[bibr53-08445621251396991] Wynn M. Garwood-Cross L. (2024). Reassembling nursing in the digital age: An actor-network theory perspective. Nursing Inquiry, 31(4), e12655. 10.1111/nin.12655 38941564

