# Supplementary material for: The Effects of an Online Patient Portal on Nurses’ and the Health Care Team's Work in an Outpatient Oncology Setting: A Qualitative Study
Source: Can J Nurs Res. 2025 Nov 24;58(2):101–13. doi: 10.1177/08445621251396991 (PMC12989629; doi:10.1177/08445621251396991)
Supplement: sj-docx-1-cjn-10.1177_08445621251396991 - Supplemental material for The Effects of an Online Patient Portal on Nurses’ and the Health Care Team's Work in an Outpatient Oncology Setting: A Qualitative Study [file sj-docx-1-cjn-10.1177_08445621251396991.docx]

**Interview Guide**

**Demographic questions:**

1. Your name (First, Last)
2. Your age
3. Employment
   1. Part time
   2. Full-time
   3. Role- nurse, clerk, MD, etc.
4. Exact position in clinics (which tumour area(s) do you work in)
5. Is English your first language?

In what capacity do you interact with Alberta Health Services Cancer Centre patients?

*In which role*

*In clinic, vs virtually, vs email, vs the portal etc.*

Do you have personal experience with AB patient portals? Which ones?- My Health Records (MHR) or MyAHS Connect (MAC) or both? As a patient or as a family member?

**Patient Reported Outcome Measures and clinic workflows**

*[Please note headings are for PA’s organization only, questions will not necessarily be asked in this order and headings will not be read aloud to participants.]*

In your work at the Alberta Health Services, do you work with patients who use MAC? MHR? How do you know which one the patient uses?

Can you describe a typical situation when a patient comes to the clinic to see you and this patient uses a MAC. How does this visit look? Please describe in detail – what forms you use, what Qs you ask, what does patient say …

Are all visits like this when a patient uses MAC? Or are there unusual meetings when a patient viewed information in MAC and asks you something …

Please tell me about the MyAHS Connect patient portal in relation to your work

Has patients pre-viewing their information on their portal changed your in-clinic interactions with them?

Do you use this form often in clinic **Show participant a physical copy of My Symptom Report form while asking question** Tell me how you use this form for patients who do not use the portals vs who do use the portals.

Do you notice patients are filling out their My Symptom Report (MSR) questionnaires through the portal before their appointments?

If a patient does fill in their MSR questionnaire through the portal prior to their follow up, what do you do with this information?

*Do you look at the MSR prior to seeing the patient?*

How does a patient pre-filling out their MSR questionnaire change your interaction with the patient? Does it change your clinic workflow?

*Do you feel your clinic workflow changes without physical copies of MSRs?*

Do you use the computer in the clinic room while you are interacting with patients?

Have you noticed your use of the computer in the room changing since the implementation of MyAHS Connect and Connect Care?

**Nursing specific**

Have you ever been assigned to the role of In-Basket management? Can you describe what this role is like for you?

*Do you think patients understand who is reading these messages when they send them in?*

Do you work in Telephone Triage? Has MyAHS Connect altered your phone call interactions with patients in TT?

What has worked well and what didn’t?

**Clerical specific**

How has the presence of the MyAHS Connect portal and Connect Care altered your processing of requisitions?

Do you work in Telephone Triage? Has MyAHS Connect altered your phone call interactions with patients in TT?

Were you ever tasked with giving patients information on how to sign up for MyAHS Connect at the check-in desk? Were you given any training on the sign-up process?

What worked well and what didn’t?

**Physician specific**

How do you feel about patients accessing results to tests you have ordered through their portal?

Do you feel presence of the MyAHS Connect patient portal changed the quantity of in-person or virtual follow ups required with patients?

What worked well and what didn’t?

**Health care provider portal perspectives**

Do you feel the presence of the MyAHS Connect patient portal changed your workflows? If yes, in what ways? Please describe and give examples.

Is there a certain demographic or type of patient you observe utilizing the portal more or less?

*Such as younger versus older patients, patients of a certain tumour group type, patients of a certain ethnicity, patients of a certain education level?*

Do you observe family members accessing the patient’s portal on their behalf?

Have patients expressed to you any specific reasons for not wanting to use the portal?

What has been your experience of patients accessing their tests results prior to their clinic visits?

Do you encourage patients to look at their test results through the portal? How do you do this? What do you say exactly?

*Do you prepare them for what to do if test results are abnormal?*

Are there any features you wish the portal has that it does not? Are there any features the portal does have that you wish it did not?

Do you think you feel similarly about MyAHS Connect compared to your colleagues? What have you observed your colleagues saying about patient use of the portal?

Have you ever had a patient suffer adverse outcomes due to their use of MyAHS Connect?

*For example, patients cancelling their own appointments.*

Have you observed a patient catching something that was missed due to the presence of MyAHS Connect?

*For example- a patient noticing they did not get booked in for a follow up appointment or have a scan ordered pre-follow up etc.*

Do you feel that MyAHS Connect has changed the quantity of work that you must do?

Where are all the places you look for patient information on Connect Care?

**Cancer disease management**

Do you feel the presence of the MyAHS Connect portal has changed anything for the patients? For example, the responsibility for taking initiative or self-management? What have you observed specifically?

**eHealth literacy**

Do you observe that patients understand how to navigate the portal and understand the information that they are reading on the portal?

Have you ever experienced patients not understanding information on their portal? What happened then?

**Patient communication with health care team**

Do patients often ask you questions about MyAHS Connect or the MyChart app?

Where are all the places you click to look for patient messages in connect care?

Do you ever directly message patients through the portal?

Do you tell patients they can electronically communicate with the health care team through the portal?

*Do you ever initiate contact with a patient electronically via the portal?*

Have you ever encouraged a patient to upload a photo using MyAHS Connect?

*Did you know this feature existed? If not, do you think it would be useful in your practice?*

Has there been clear instructions or rules on how you are allowed to interact with patients through the portal?

**Communication between health care members**

Has the presence of Connect Care or MyAHS Connect changed the way you interact with your colleagues? How so?

*For nurses- do you ever forward direct patient messages to physician staff?*

**Education**

Was the MyAHS Connect portal discussed during your orientation to Connect Care?

Have you ever had the opportunity to see what the MyAHS Connect portal looks like?

Have you ever seen what the MyChart app looks like?

Do you feel familiar with what features the MyAHS Connect portal offers?

Do you encourage patients to sign up for and utilize the MyAHS Connect patient portal?

*Has management or anyone else encouraged you to do this?*

If a patient asks you how to sign up for the portal what do you tell them?

**Any final thoughts that we have not covered that you would like to share with me today?**

*[Researcher will thank a participant at the end of the interview and inform them that a $40 gift card will be sent via email.]*
